# Imbalanced IgA-IgG class switching recombination: a novel mechanism of gut immune dysregulation in inflammatory bowel disease

**DOI:** 10.3389/fimmu.2026.1836986

**Published:** 2026-08-03

**Authors:** Ke Chen, Yangqiao Qing, Wei Gao, Wen Feng, Jun Tian, Jing Wang

**Affiliations:** 1 Songjiang Hospital Affiliated with Shanghai Jiao Tong University School of Medicine, Shanghai, China; 2 Hospital of Stomatology, Guanghua School of Stomatology, Guangdong Provincial Key Laboratory of Stomatology, Sun Yat-Sen University, Guangzhou, China

**Keywords:** class switch recombination (CSR), humoral immunity, IBD - inflammatory bowel disease, IgA, IgG

## Abstract

Inflammatory bowel disease (IBD) is a chronic intestinal inflammatory disorder with significant disease burden that markedly impairs patients’ quality of life. The pathogenesis of IBD is closely associated with hyperactive immunopathological responses. Emerging evidence has established humoral immunity as a crucial contributor to IBD development. Class switch recombination (CSR), the critical process enabling B cells to produce different antibody isotypes, plays a pivotal role in humoral immunity. While healthy intestinal mucosa predominantly contains immunoglobulin A (IgA) that maintains gut homeostasis, immunoglobulin G (IgG) becomes the dominant isotype during IBD and drives chronic inflammation. Therefore, understanding the signals and mechanisms governing antibody CSR may provide deeper insights into IBD pathogenesis. Herein, we review B cell activation pathways, the distinct roles of IgA and IgG in intestinal immunity, CSR-regulating signals, and recent discoveries in the context of IBD, with the aim of identifying novel therapeutic opportunities.

## 1 Introduction

Inflammatory bowel disease (IBD) is a chronic, recurrent inflammatory disorder of the gastrointestinal tract, primarily classified into ulcerative colitis (UC) and Crohn’s disease (CD) (1). The exact pathogenesis of IBD remains incompletely elucidated, but an aberrant immune response to the gut microbiota is considered a key driver of IBD. Previous research has largely focused on T cells and their production of pro-inflammatory cytokines, such as TNF-α, IFN-γ, IL-12, and IL-23. Consequently, a series of biologic agents targeting these cytokines have been applied in clinical treatment. Although the application of biologics has led to significant therapeutic advances, a large number of patients still face primary or secondary treatment failure in clinical practice. This therapeutic bottleneck compels us to reexamine humoral immunity in the gut.

As early as the 1980s, Sieber et al. observed a significant increase in the number of spontaneously secreting immunoglobulin cells in the peripheral blood of patients with active CD (nearly 10 times that of normal controls) (2), suggesting widespread *in vivo* polyclonal B-cell activation during the course of IBD. Although early basic and clinical studies had already suggested abnormal activation of humoral immunity in IBD, the role of humoral immunity in IBD was not fully recognized for some time because a clinical study found that rituximab (an anti-CD20 antibody) showed no significant effect on inducing remission in patients with moderate active UC who were unresponsive to oral steroids (3). In fact, rituximab-mediated depletion of CD20^+^ B cells does not affect intestinal-resident plasma cells (4). With further research, the role of humoral immunity in local intestinal immunity has gained increasing attention. B cells initially secrete low-affinity IgM, but under the coordinated action of multiple signals, class switch recombination (CSR) is induced, ultimately generating different antibody types to combat various pathogens (5). Gut-associated lymphoid tissues (GALT, including Peyer’s patches and isolated lymphoid follicles) and mesenteric lymph nodes (MLNs) are the primary sites where B cells encounter antigens and undergo CSR. CSR is divided into two pathways: T-cell-dependent (TD) and T-cell-independent (TI). The former relies on germinal centers (GCs) and occurs primarily in Peyer’s patches and MLNs, while the latter is mainly confined to isolated lymphoid tissues lacking typical GCs (6, 7). These plasma cells, which differentiate and mature through different pathways, subsequently migrate to the lamina propria of the intestinal mucosa, where they reside and secrete antibodies. In healthy intestinal mucosa, immunoglobulin A (IgA) predominates among antibodies to promote commensalism with gut microbiota and maintain intestinal homeostasis. However, in IBD patients, the more pro-inflammatory IgG becomes predominant (8). This suggests that the signaling of antibody CSR may be altered in IBD.

A growing body of evidence suggests that the intestinal pathological microenvironment in IBD can “domesticate” late-stage B cells *in situ*, ultimately causing them to deviate from their normal physiological differentiation pathway (9). In IBD, first, intestinal pro-inflammatory factors IgG CSR and inhibit the formation of physiological IgA; second, the inflammatory environment blocks the sequential conversion pathway from IgM to IgG1 to IgA, causing intermediate IgG1^+^ B cells to accumulate in the lamina propria and secrete pro-inflammatory antibodies; finally, in terms of subclass distribution, pro-inflammatory IgG1 and IgG3 increase, while the conversion of protective IgA2 is impeded. A better understanding of intestinal B cells and the key mechanisms of intestinal IgA and IgG CSR will enhance our knowledge of IBD pathogenesis and therapeutic development. This review summarizes current knowledge on B cell activation, the roles of IgA and IgG in intestinal immunity, CSR signaling pathways, and recent discoveries in the context of IBD.

## 2 Intestinal B-cell activation and CSR initiation

**Figure 1:**
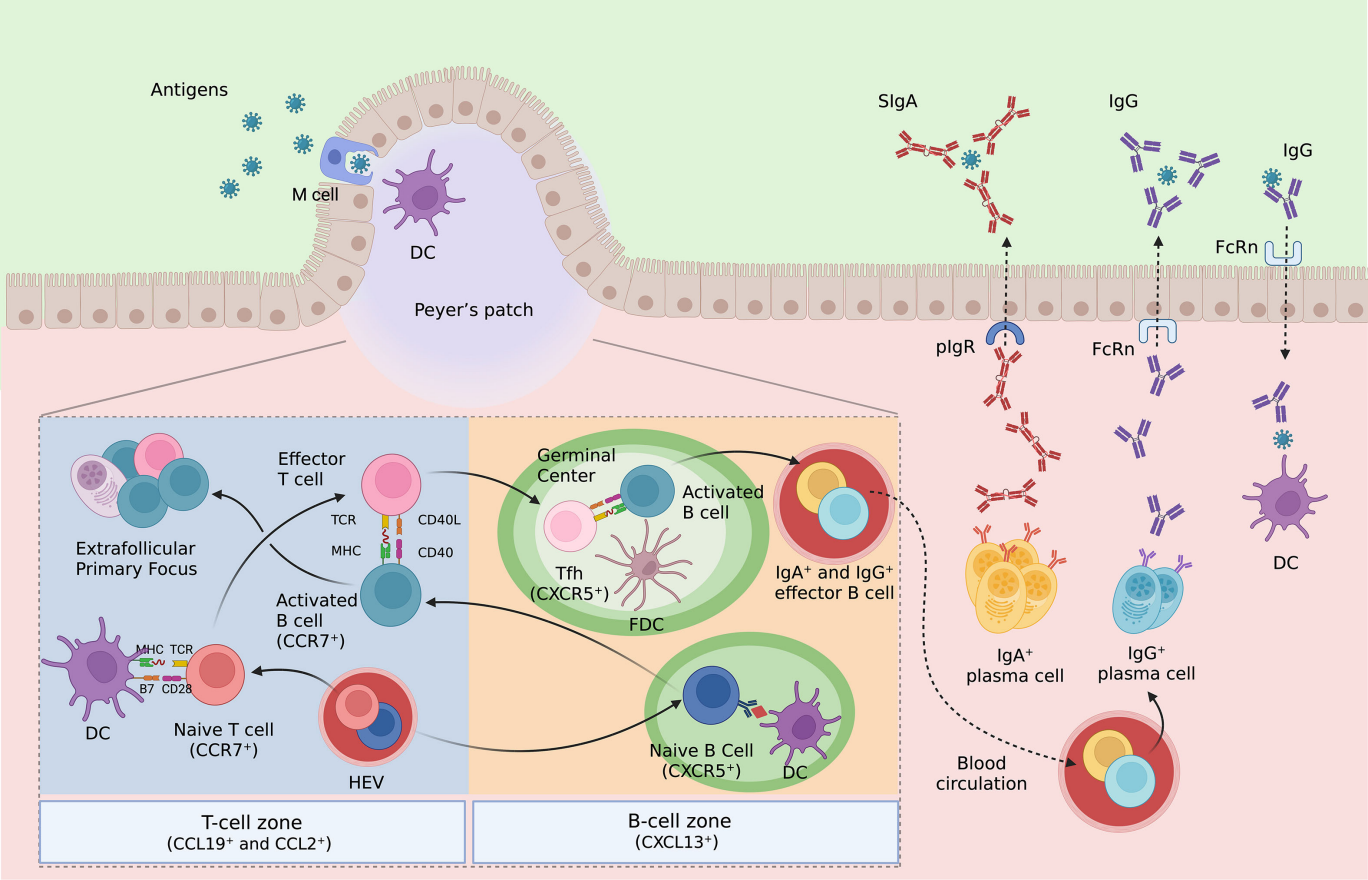
T cell-dependent (TD) antibody production. This image illustrates cell migration and cell-cell interactions during T-cell-dependent antibody production in gut-associated lymphoid tissue (GALT). Luminal antigens are taken up by specialized M cells in the follicle-associated epithelium and delivered to subepithelial dendritic cells (DCs) within Peyer’s patches. Naive B and T cells enter Peyer’s patches through high endothelial venules (HEVs). CXCR5-expressing naive B cells migrate to B cell follicles (where the CXCR5 ligand CXCL13 is expressed) and, after initial activation by DCs, upregulate CCR7, enabling their migration toward T cell zones (where CCR7 ligands CCL19 and CCL21 are expressed). Concurrently, DCs present processed antigens via MHC to naive T cells, which differentiate into effector T cells. Activated B cells and effector T cells interact at the T-B cell interface. A subset of T cells differentiates into CXCR5^+^ follicular helper T cells (Tfh) and enters lymphoid follicles, while B cells that have undergone T-B interactions return to the follicles, where they proliferate extensively and form germinal centers. A small number of B cells may establish extrafollicular foci in the medulla, generating short-lived plasma cells, though these are limited in scale. Within germinal centers, Tfh cells and follicular dendritic cells collaboratively mediate antibody class switch recombination (CSR) and differentiation of B cells, ultimately generating IgA^+^ and IgG^+^ effector B cells. These effector B cells enter the circulation and, guided by integrins and chemokines, traverse intestinal HEVs to home to the lamina propria, where they mature into IgA^+^ and IgG^+^ plasma cells and secrete antibodies. In the intestinal lamina propria, dimeric IgA binds to the polymeric immunoglobulin receptor (pIgR) on the basolateral surface of epithelial cells, forming secretory IgA (SIgA) that is released into the intestinal lumen. SIgA maintains gut homeostasis by neutralizing pathogens, anchoring beneficial bacteria, and reducing pro-inflammatory signals from commensals. IgG, meanwhile, undergoes bidirectional transport via the neonatal Fc receptor (FcRn): on one hand, it transports lamina propria-derived IgG to the lumen to exert neutralization, opsonization, complement-dependent cytotoxicity, and antibody-dependent cellular cytotoxicity; on the other hand, it mediates the transcytosis of luminal IgG-antigen complexes, which are internalized and presented by DCs to activate both cellular and humoral immune responses.

Under physiological conditions, immature B cells migrate specifically to GALT (such as Peyer’s patches and isolated lymphoid follicles) and MLN via high endothelial venules (HEVs) or afferent lymphatic vessels (10) (**Figure 1**). Under the precise regulation of chemokine axes (such as CXCR5/CXCL13 and CCR7/CCL19/CCL21), B cells acquire antigens presented by stromal or myeloid cells in the follicular zone (thereby initiating the first signal required for activation and CSR) and migrate to the T-B cell interface (11–14). Here, B cells engage in bidirectional interactions with follicular helper T cells (Tfh), receiving a second signal provided by CD40L and specific cytokines. They subsequently enter the GC or extrafollicular medullary foci, where they undergo high-frequency somatic mutation, antibody affinity maturation, and CSR, ultimately differentiating into plasma cells that home to the intestinal lamina propria, constituting the TD antibody response (15–17). In intestinal regions lacking GCs (such as isolated lymphoid tissues), microbial antigens such as lipopolysaccharides can directly induce B cells to mount a TI antibody response and CSR without T-cell assistance (6, 7). These two pathways collectively maintain the non-inflammatory humoral immune homeostasis of the intestinal mucosa.

However, in the pathological progression of IBD, antigen overload and microenvironmental disruption caused by mucosal barrier damage lead to dysregulation of the TD and TI activation pathways in B cells, thereby altering the normal direction of CSR. At this stage, the previously dominant protective IgA (especially the highly stable IgA2) shifts significantly toward pro-inflammatory IgG (such as IgG1) and the short-lived IgA1 subclass. This CSR abnormality contributes to mucosal damage and increases the risk of IBD-related colorectal cancer.

## 3 Effector functions of intestinal IGA and IGG

### 3.1 Effector function of IgA

Under physiological conditions, the lamina propria of the human intestinal mucosa consists primarily of IgA-secreting plasma cells, which maintain immune tolerance toward the intestinal microbiota (18). Plasma cells in the lamina propria of the normal human small intestine are heterogeneous and can be classified into three subsets based on the expression of CD19 and CD45: the CD19^+^CD45^+^ subset, the CD19^−^CD45^+^ subset, and the CD19^−^CD45^−^ subset. All three subsets highly express plasma cell markers, and the vast majority are IgA-secreting plasma cells. Among these, the CD19^+^ plasma cell subpopulation exhibits higher IgA secretory activity *in vitro*. Furthermore, these intestinal plasma cells have a relatively long overall lifespan. These long-lived and heterogeneous IgA plasma cells play a crucial role in maintaining intestinal mucosal homeostasis and preventing the translocation of commensal bacteria (19).

As the most abundant immunoglobulin isotype in the human gut (18), humans have two IgA subclasses: IgA1 and IgA2, unlike immunodeficient mice, which possess only one IgA type. Structurally, the hinge region of the IgA2 heavy chain is shorter than that of IgA1, conferring greater resistance to microbial proteases, which may enhance its durability in the colon (20). The distribution of IgA^+^ plasma cells varies significantly across different intestinal segments. The small intestinal lamina propria harbors the highest proportion (approximately 80%) of IgA^+^ plasma cells, whereas their abundance in the colonic lamina propria is considerably lower (21). The ratio of IgA1^+^ to IgA2^+^ plasma cells also exhibits regional variation, shifting from 3:1 in the proximal small intestine to 1:3 in the colon (22). Beyond structural and distributional differences, IgA1 and IgA2 exhibit distinct cellular dynamics and reactivity profiles. Recent studies have revealed that IgA1 is primarily enriched within the CD19^+^ short-lived plasma cell subset and displays a higher turnover rate. In contrast, IgA2 is predominantly found within the CD19^−^ long-lived plasma cell subset, suggesting that IgA2 constitutes the core of long-term immune memory in the gut (23). In addition, IgA1 exhibits broader polyreactivity, enabling it to bind a wide array of commensal bacteria, while IgA2 shows a more restricted reactivity towards specific microbial taxa and exerts more potent anti-inflammatory protective effects in maintaining intestinal homeostasis (23).

Intestinal IgA exists primarily as dimers linked by J-chains and is transported into the intestinal lumen via the polyimmunoglobulin receptor (pIgR), where it forms secretory IgA (SIgA) (24) (**Figure 1**). The intestine represents one of the largest mucosal surfaces in the human body. The intestinal mucosal immune system must produce protective immunity against pathogens while maintaining tolerance to harmless food antigens and commensal microorganisms. SIgA can neutralize toxins and pathogens without activating the complement system or triggering inflammation, thereby playing a crucial role in maintaining intestinal homeostasis. First, SIgA can specifically bind to beneficial commensal bacteria (such as Bacteroides) and anchor them within the mucus layer, promoting a mutually beneficial symbiosis between the host and microorganisms by downregulating the expression of pro-inflammatory epitopes and signals in these commensal bacteria (25, 26). Second, IgA possesses a central function in regulating the composition of the gut microbiota and maintaining symbiotic balance (27). Furthermore, intestinal IgA can selectively recognize and coat potential pathogenic or opportunistic pathogens, confining them within the intestinal lumen through immune exclusion to prevent their trans-epithelial migration, thereby promoting their transition to a harmless symbiotic state (28). However, in the pathological state of IBD, high levels of IgA-coated bacteria become an immunological marker of pro-inflammatory bacteria. Using IgA-SEQ technology, Palm et al. found that the proportion of IgA-coated bacteria in the intestines of IBD patients was significantly increased. These IgA^+^ bacterial populations can invade the colonic mucus layer; when transplanted into germ-free mice, they exacerbate DSS-induced colitis. This suggests that, in the pathological context of IBD, although the host’s robust IgA response to susceptible bacterial populations fails to convert them into a harmless symbiotic state, this specific IgA response can serve as a functional biomarker for identifying gut microbial groups that influence disease susceptibility or severity (29).

### 3.2 Effector function of IgG

Compared to secretory IgA, which has anti-inflammatory properties, IgG exhibits stronger pro-inflammatory characteristics. It primarily clears antigens by neutralizing them, mediating Fcγ receptor (FcγR)-dependent opsonized phagocytosis, and activating the classical complement pathway and antibody-dependent cell-mediated cytotoxicity (ADCC) (30, 31).

Human IgG is divided into four subclasses, each with distinct characteristics in terms of abundance and function. Among these, IgG1 is the most abundant, followed by IgG2, IgG3, and IgG4 (32). Functionally, IgG1 and IgG3 exhibit powerful effector functions, including antibody-dependent cellular cytotoxicity and complement-dependent cytotoxicity. In contrast, IgG2 and IgG4 responses are typically weaker, probably due to their short and rigid hinge regions that impair antibody flexibility (33), which can affect affinity for FcγRs and complement. IgG3 is a strongly pro-inflammatory antibody usually produced in the early stages of infection, yet its relatively short half-life may help limit excessive inflammatory responses (32).

Although IgA predominates in the healthy gut, scRNA sequencing has revealed the presence of low levels of IgG^+^ plasma cells in non-infected human colon (34). Koch et al. demonstrated commensal-reactive IgG2b and IgG3 responses in murine mesenteric lymph nodes and Peyer’s patches (35). Maaser C et al. found that intestinal mucosal defense against Citrobacter rodentium in mice depends on IgG, without requiring the production or trans-epithelial transport of secretory IgA or IgM (36). A portion of the IgG in the mouse intestine is derived from the mother; it serves to coat pathogens in the intestinal lumen to reduce colonization (37) and protects against allergic intolerance to food (38). Therefore, IgG in the healthy gut may play an important role in maintaining homeostasis. In the human intestine, IgG exerts its functions through bidirectional transport mediated by the neonatal Fc receptor (FcRn) in intestinal epithelial cells (**Figure 1**). FcRn not only transports IgG from the lamina propria to the intestinal lumen, but also transports IgG-antigen immune complexes from the intestinal lumen back to the lamina propria to activate a local immune response that eliminates the antigen (39, 40). Collectively, low levels of commensal-reactive IgG exist in the healthy human gut. They may maintain controlled local inflammation to prevent pathogen translocation and enhance epithelial barrier function, thereby preserving intestinal homeostasis. However, uncontrolled elevation of IgG levels can promote mucosal inflammation and ultimately contribute to the development of immune disorders such as IBD.

## 4 Mechanisms of CSR in the gut

Through CSR, the human humoral immune system generates five classes of antibodies: IgM, IgA, IgG, IgE, and IgD. In its absence, humans and other mammals would only produce IgM and IgD (41), severely limiting the effectiveness of humoral immune responses against infections.

### 4.1 The process of CSR

**Figure 2:**
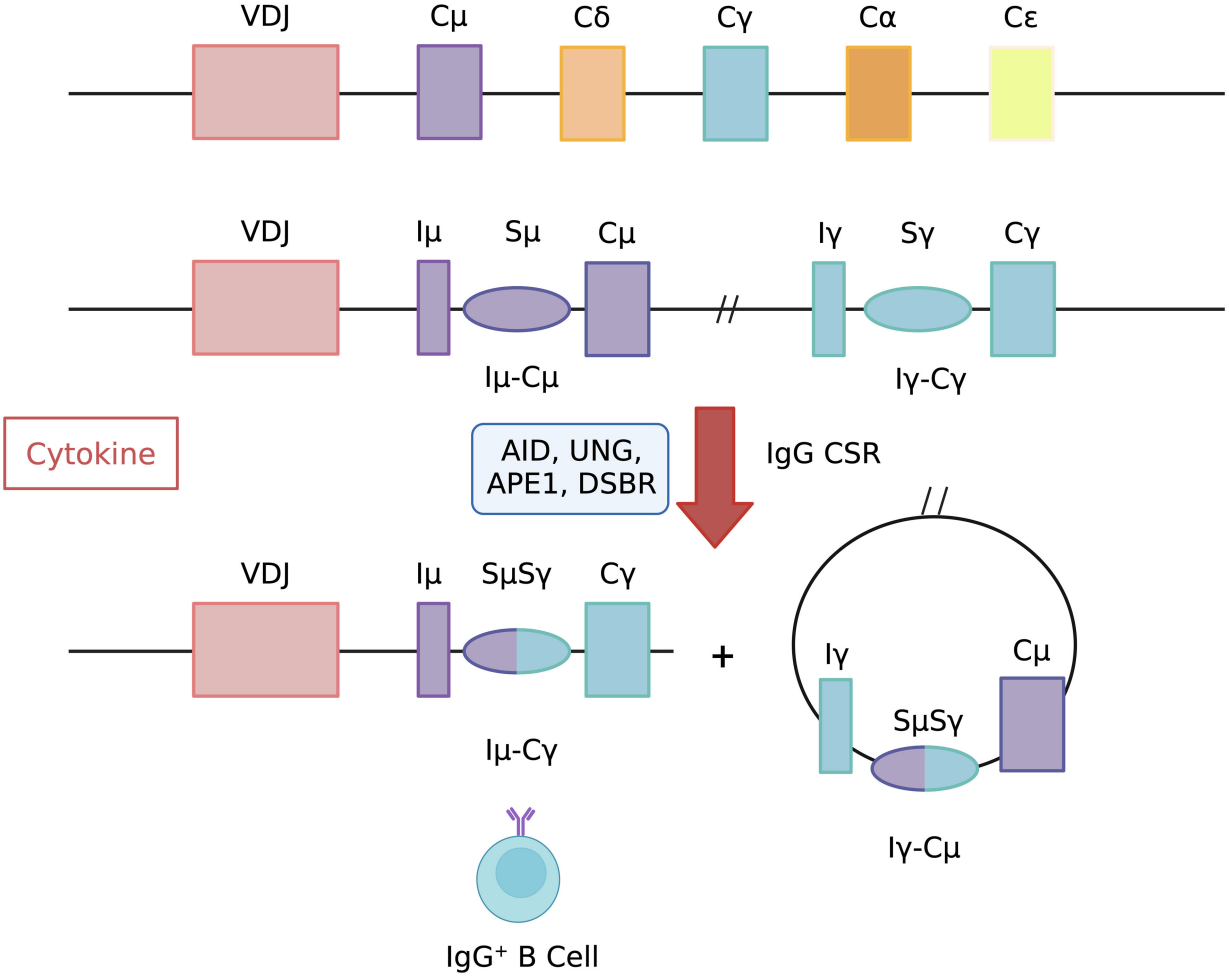
The process of IgG CSR. In the antibody gene locus of B cells, the constant region (C region) downstream of the VDJ gene segments is arranged in a specific order: Cμ, Cγ3, Cγ1, Cα1, Cγ2, Cγ4, Cϵ, and Cα2, which encode IgM, IgG3, IgG1, IgA1, IgG2, IgG4, IgE, and IgA2, respectively. Each C region is preceded by an intervening region (I region) and a switch region (S region). Under the induction of the cytokine microenvironment, double-strand breaks occur between the Sμ region and the target Sγ region. Subsequently, enzymes such as activation-induced cytidine deaminase (AID), uracil-DNA glycosylase (UNG), and AP endonuclease 1(APE1) generate multiple nicks in the single-stranded DNA of the S regions. Through the double-strand break repair (DSBR) mechanism, the broken Sμ and Sγ chains align and join, fusing to form the SμSγ transition region. The intervening DNA fragment is excised, forming circular excision products and Iμ-Cγ transcripts, thereby completing class switch recombination (CSR). This process brings the VDJ segment adjacent to the target C region, enabling transcription driven by the promoter and ultimately leading to the expression of IgG-class antibodies.

The immunoglobulin heavy chain gene in B cells contains a unique VDJ segment, downstream of which the constant (C) region genes are sequentially arranged as Cμ, Cγ3, Cγ1, Cα1, Cγ2, Cγ4, Cϵ, and Cα2, corresponding to antibody isotypes IgM, IgG3, IgG1, IgA1, IgG2, IgG4, IgE, and IgA2, respectively (**Figure 2**). In front of the C region, the intervening region (I region) and the transition region (S region) are arranged. Taking IgG CSR as an example, B cells initially produce low-affinity IgM. Under the influence of the cytokine microenvironment, the double chain of the Sμ and Sγ regions breaks into a single strand. Activation-induced cytidine deaminase (AID), uracil-DNA glycosylase (UNG), and AP endonuclease 1 (APE1) collaboratively generate gaps in the single strand of S regions. The double-strand break repair (DSBR) machinery then mediates the joining of two S regions with concomitant excision of the intervening DNA loop between Sμ and Sγ, ultimately forming the Iμ-Cγ germline transcript. This process places the specific transcribed region downstream of the VDJ base segment, and then the promoter (P) upstream of the VDJ segment drives the transcription process, ultimately producing IgG (42–44).

AID is a single-strand-specific DNA mutator that catalyzes the deamination of cytosine to uracil in the S region DNA (45, 46). These uracil bases are then excised by UNG (47, 48) and APE1, generating single-strand breaks (SSBs) (49). These breaks, when closely spaced on complementary strands or processed by the mismatch repair system, form double-strand breaks (DSBs). Finally, the DSBR machinery mediates the recombination and ligation of the two targeted S regions, excising the intervening DNA loop to complete the switch from IgM to the target isotype (5). Thus, AID plays a crucial role in CSR by introducing SSBs into S regions and converting them into the DSBs required for CSR.

For a long time, this single-step transition from IgM to target isotypes has been regarded as the standard model for CSR. However, a recent study utilizing the S1PR2-CreERT2 lineage tracing model revealed that a significant portion of IgA plasma cells in the mouse intestine do not derive directly from IgM, but instead originate from B cells that previously expressed IgG1 (50). This was further evidenced by the identification of unique Sμ-Sγ1-Sα junctional circles in the genome of IgA B cells, a process termed “sequential class switch recombination” (sequential CSR) from IgM to IgG1 and then to IgA. Functionally, IgA cells generated through sequential CSR exhibit higher levels of somatic hypermutation and produce antibodies with superior affinity compared to those derived from direct CSR. This suggests that precise immune responses against specific pathogens or dietary antigens are highly dependent on the IgG1 intermediate stage. Furthermore, B cell repertoire analysis of human upper airway samples has demonstrated that this sequential switching mechanism is also conserved in the human mucosal immune system. These findings indicate that IgG1 cells are not only participants in inflammation but also essential precursors for high-quality intestinal IgA. Notably, the scope of sequential switching extends beyond the inter-isotype transition from IgG1 to IgA and is also observed between human intestinal IgA subclasses. Research has identified that a fraction of IgA2 in the human gut does not derive directly from IgM, but instead originates from IgA1 precursor cells through a multi-step pathway of IgM→IgA1→IgA2 (23). These findings collectively indicate that multi-stage sequential switching is a core mechanism for the mucosal immune system to generate high-precision antibodies, playing an irreplaceable role in maintaining intestinal homeostasis. However, when this sequential switching mechanism is disrupted or stalled during disease, B cells may become trapped in the pro-inflammatory IgG1 stage, leading to the depletion and dysregulation of the mucosal humoral immune barrier.

### 4.2 The signals that induce and determine CSR in the gut

Two types of signals are required for CSR to occur in intestinal B cells. CD40 and TLR signaling pathways serve as crucial primary signals that induce the production of AID (5). Cytokines produced by T cells and DCs are secondary signals for CSR, directing the switch from IgM to different antibody isotypes.

#### 4.2.1 Primary signals inducing CSR

During the TD response, antigen-activated B cells migrate to the T-B cell border zone, where CD40 on B cells binds to CD40L on T cells, further activating B cells and initiating GCs formation along with subsequent reactions. Deficiencies in CD40L or CD40 lead to hyper-IgM syndrome in humans and significantly reduce IgG, IgA, and IgE levels in mice (5). In fact, murine studies demonstrate that CSR is strongly induced during early T-B cell interactions (51). Marshall et al. further revealed that extrafollicular B cell activation induces AID expression and CSR within the first few days of B cell activation (52), just after they enter the edge of the T-cell zone. These findings collectively demonstrate the crucial role of T cells and surface CD40L molecules in CSR induction. In TI responses, while intestinal B cells deficient in Atg5 retain immunoglobulin production and CSR capacity, their impaired responsiveness to TLR stimulation compromises antibody secretion. Atg5-knockout mice exhibited reduced spontaneous IgA secretion by B cells in gut-associated tissues (MLNs, PPs, and LP) (53). Following DSS-induced colitis, these B cells were still unable to secrete IgA and the intestinal inflammation was more severe (53). These evidences confirms that primary signals serve as fundamental determinants for antibody CSR in both TD and TI immune responses.

#### 4.2.2 Secondary signals regulating IgA CSR

**Figure 3:**
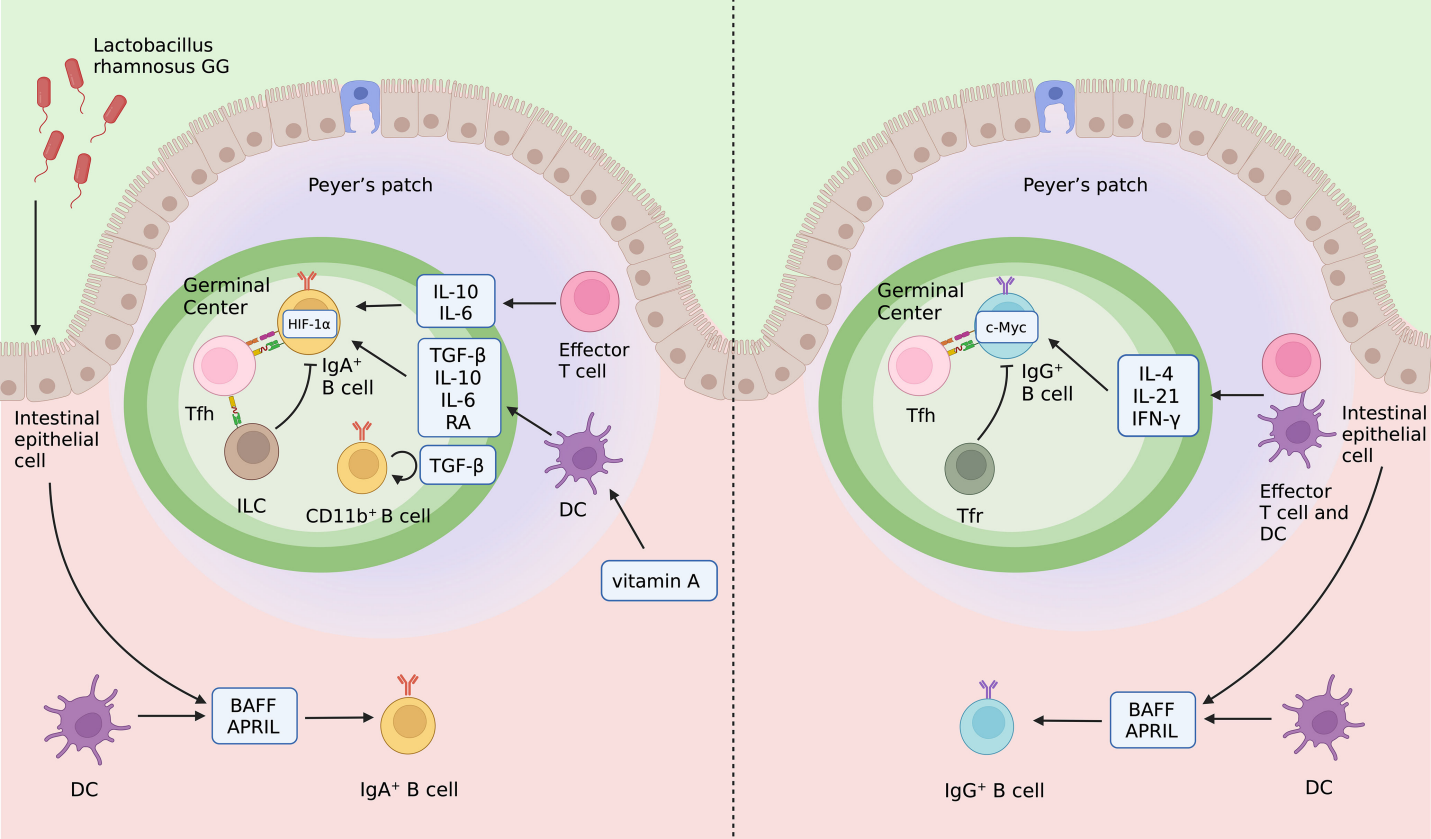
Modulation mechanisms of IgA and IgG CSR in the intestine. Left panel (Modulation mechanisms of IgA CSR): In Peyer’s patches, multiple immune cells (effector T cells, DCs, etc.) promote T cell-dependent IgA class switch recombination (CSR) through secretion of IL-10 and IL-6. TGF-β serves as the core factor for IgA induction. Retinoic acid (RA), a vitamin A metabolite produced by DCs, enhances IgA production by stimulating DCs to release TGF-β. CD11b^+^ B cells facilitate their own IgA CSR via autocrine TGF-β signaling. HIF-1α supports IgA generation by enhancing glycolytic metabolism. BAFF and APRIL (expressed on DCs and intestinal epithelial cells) induce T cell-independent (TI) IgA CSR. Lactobacillus GG can promote IgA CSR by upregulating APRIL expression in intestinal epithelial cells. ILC3s suppress colonic IgA^+^ B cell generation through antigen presentation to follicular helper T cells (Tfh). Right panel (Modulation mechanisms of IgG CSR): Current research on IgG CSR primarily focuses on splenic or systemic B cells, and these findings provide valuable references for exploring intestinal IgG CSR mechanisms. In mice, IL-4 and IL-21 have been demonstrated to cooperatively promote IgG1 CSR; IL-17A and IL-21 drive IgG2a/IgG3 and IgG1/2b sCSR, respectively; IFN-γ can induce IgG2a production. c-Myc is essential for IgG1 switching and may influence IgG2b/2c conversion through metabolic regulation. BAFF and APRIL can mediate TI IgG1 CSR. Follicular regulatory T cells (Tfr) suppress Tfh-mediated IgG secretion.

During B cell activation, immune cells including T cells, DCs, and Tfh cells, as well as intestinal epithelial cells, modulate the isotype of CSR through secretion of specific cytokines. In the mouse intestine, transforming growth factor beta (TGF-β), IL-10, and IL-6 have been shown to support IgA CSR (42) (**Figure 3**). TGF-β, in particular, plays a crucial role in IgA induction. Vitamin A can be oxidatively metabolized into its active metabolite retinoic acid. The latter promotes IgA production by stimulating DCs in Peyer’s patches and mesenteric lymph nodes to release active TGF-β (54). Compared to control mice, vitamin A-deficient mice exhibited more severe DSS-induced colitis (55). Gut-associated lymphoid tissues in mice are enriched with CD11b^+^ B cells, whose numbers significantly increase following DSS-induced colitis. These CD11b^+^ B cells express abundant TGF-β and exhibit highly activated phosphorylated Smad2/3 signaling, thereby promoting IgA CSR to suppress intestinal inflammation through autocrine mechanisms (56). The TNF family members B cell-activating factor (BAFF) and a proliferation-inducing ligand (APRIL), expressed by DCs and intestinal epithelial cells, can facilitate TI IgA CSR through interaction with the receptor transmembrane activator and CAML interactor (TACI) (57–59).

Intestinal IgA levels and AID expression in lamina propria have been shown to correlate with the abundance of Bacteroides fragilis species (60). p40, a Lactobacillus rhamnosus GG-derived protein, activates epidermal growth factor receptor signaling, thereby upregulating APRIL expression in murine intestinal epithelial cells and subsequently enhancing IgA class switching and production in gut B cells (both *in vivo* and *in vitro*) (61). Furthermore, Bacteroides species including B. faecis, B. caccae, and B. acidifaciens have been demonstrated to promote intestinal IgA production and AID expression in mice (60). The precise mechanisms underlying these microbiota-mediated effects require further investigation.

Several factors can epigenetically promote IgA CSR. Transcriptional regulatory protein hypoxia-inducible factor 1-alpha (HIF-1α) enhances IgA CSR by increasing glycolytic metabolism in intestinal B cells. Specifically, HIF-1α increases histone H3K27 acetylation in the Sα region through acetyl-coenzyme A, thereby facilitating IgA CSR. Genetic ablation of HIF-1α in mice impairs the differentiation of IgA^+^ B cells in MLNs and exacerbates DSS-induced colitis. Either HIF-1α overexpression or acetyl-coenzyme A supplementation can increase intestinal IgA secretion in mice and provide protection against DSS-induced colitis (62).

In contrast to factors that promote IgA CSR, only a few studies have been conducted on negative regulators of IgA CSR to date. In GCs, Tfh cells cooperate with follicular dendritic cells to initiate GC reactions in B cells. Felipe Melo-González et al. discovered that group 3 innate lymphoid cells (ILC3s) can reside in the follicles of murine MLNs and present antigens to Tfh cells, thereby suppressing GC reactions and the generation of IgA^+^ B cells in the colon (63). As critical regulators of colonic IgA production, ILC3s support the survival of commensal microorganisms in the gut and play an essential role in maintaining intestinal homeostasis.

#### 4.2.3 Secondary signals regulating IgG CSR

The cytokine-mediated signals that directly promote intestinal IgG CSR remain poorly understood, as most studies on IgG CSR have been conducted (58). *In vitro*, IL-4 and IL-21 cooperatively promoted TI IgG1 CSR in mouse splenic B cells (64) (**Figure 3**). Subsequently, Jason S. Weinstein et al. demonstrated that IL-4 and IL-21 also synergistically induce IgG1 CSR in GC B cells *in vivo* (65). Similarly, another study revealed that IL-17A and IL-21 promote IgG2a/IgG3 and IgG1/IgG2b CSR, respectively, in murine splenic B cells *in vitro*. IFN-γ can drive CSR to IgG2a in both splenic and lymph node B cells *in vivo* and *in vitro* (66). Both BAFF and APRIL induce TI IgG1 and IgA CSR in mouse splenic B cells *in vitro* (58). Notably, APRIL-mediated CSR depends exclusively on TACI receptor signaling in B cells, whereas BAFF utilizes both TACI and BAFF-R for CSR induction. However, TACI signaling is indispensable for BAFF-induced IgA CSR (58).

As mentioned previously, HIF-1α promotes IgA CSR by enhancing glycolytic metabolism in intestinal B cells (67). The transcription factor c-Myc represents another key regulator of glycolytic metabolism. Studies using murine splenic B cells *in vitro* have demonstrated that c-Myc is essential for IgG1 CSR (68). Furthermore, c-Myc-dependent metabolic regulation may serve as an important modulator of IgG2b and IgG2c CSR (62).

Regarding inhibitory factors of IgG CSR, a study by Wang et al. revealed that in contrast to Tfh cells which provide co-stimulatory signals and cytokines to activate B cells, circulating follicular regulatory T (Tfr) cells can suppress Tfh-mediated IgG CSR and secretion (69). Cytotoxic T-lymphocyte-associated protein 4 (CTLA-4) is essential for Tfr-mediated suppression, as CTLA-4 blockade significantly attenuates Tfr-dependent IgG inhibition. It should be noted that this study exclusively used peripheral blood mononuclear cells, and whether Tfr in the intestine plays the same role requires further investigation.

Currently, most studies on IgG CSR have employed splenic or circulating B cells, but whether these findings can be extrapolated to intestinal B cells remains unclear. Nevertheless, these investigations provide valuable insights for future studies on intestinal CSR. Importantly, antibody CSR represents a precisely regulated physiological process resulting from the dynamic interplay between promoting and inhibitory factors. In the context of IBD pathology, this process is easily disrupted: when the intestinal barrier is compromised, leading to a massive invasion of commensal bacteria into the lamina propria, pro-inflammatory cytokines such as IL-4, IL-21, IL-17A, and IFN-γ abnormally surge in the local microenvironment. This not only overwhelms the existing IgA-biased signaling but also disrupts the immunosuppressive barrier provided by cells such as Tfr. This pathological signaling disruption erroneously triggers isotype switching toward highly pro-inflammatory IgG subclasses, leading to uncontrolled increases in local IgG levels in the intestinal mucosa and ultimately driving the chronic mucosal inflammation characteristic of IBD. It is important to note that most current research on regulatory signals focuses on the traditional single-step CSR pathway. In contrast, the regulatory mechanisms of the emerging “continuous CSR” in the complex intestinal microenvironment remain largely unknown. This represents a critical direction for future exploration of intestinal immune regulation mechanisms.

## 5 Dysregulated CSR in IBD

Previous studies on IBD have predominantly focused on T cells and their associated cytokines. With advancing research, more and more studies have shown that humoral immunity plays an important role in the development of IBD. In IBD, the intestinal CSR network is disrupted, primarily manifesting as an ‘IgA-IgG CSR imbalance.’ This imbalance involves more than just changes in antibody levels; it encompasses quantitative biases in cell fate decisions, stagnation within switching pathways, and the dysregulation of subclass effector functions. These intertwined disruptions ultimately shift intestinal mucosal humoral immunity from an anti-inflammatory homeostatic state to a pro-inflammatory pathological state.

### 5.1 Abnormal intestinal IgA CSR in IBD

In patients with IBD, intestinal IgA levels are increased compared to healthy controls. Nevertheless, this increase in IgA fails to effectively counteract inflammation. While the rise in pro-inflammatory IgG far exceeds that of IgA, more importantly, the excess IgA itself contributes to the progression of intestinal inflammation. In the healthy intestine, intestinal mononuclear cells primarily secrete dimeric IgA, with monomers accounting for only 31%. In contrast, the proportion of monomeric IgA is significantly increased in patients with IBD, reaching 43% in CD and 53% in UC. Because monomeric IgA lacks a J chain, it cannot be transported across the epithelium into the intestinal lumen via the polyimmunoglobulin receptor (pIgR), resulting in a large amount of untransported IgA remaining in the lamina propria (70). Concurrently, the compromised intestinal barrier in IBD allows more IgA-coated bacteria to enter the lamina propria. These IgA complexes activate the receptor FcαRI on the surface of myeloid cells, such as monocytes and neutrophils, inducing the secretion of the chemokine CCL20 and other inflammatory cytokines (71). This process further recruits B cells to the inflamed areas and, under the influence of cytokines like IL-6, TNF-α, and APRIL, promotes their differentiation into IgA plasma cells. This creates a vicious cycle that generates more IgA and sustains chronic inflammation. Furthermore, there is a significant imbalance in the distribution of IgA subclasses in the intestinal mucosa of IBD patients. Under homeostatic conditions, the colon is enriched with long-lived, protective IgA2^+^ plasma cells that maintain long-term immune tolerance. However, in IBD, there is an abnormal expansion of short-lived IgA1^+^ plasma cells, while IgA2^+^ plasma cells are markedly reduced (23, 70). Because the hinge region of IgA1 is more susceptible to degradation by bacterial proteases, this subclass shift weakens the coating efficiency on the mucosal surface, leading to a decline in barrier protection. Recent studies combining spatial single-cell proteomics have shown that in the lamina propria of the intestinal mucosa in CD patients, there is an abnormal increase in early immature CD19^+^CD45^+^ plasma cells, while mature CD19^−^CD45^−^ plasma cells that secrete high levels of dimeric IgA are significantly reduced (72). This expansion of immature plasma cells and their spatial competition with mature plasma cells represents a key cellular dynamic mechanism underlying the local mucosal deficiency of SIgA.

The aforementioned IgA tissue accumulation and subclass imbalance are primarily observed in localized lesions during active disease, whereas immune regulation in unaffected regions and across different disease stages is more complex. In an early study, researchers observed that in histologically unaffected jejunal mucosa of patients with CD, both the luminal secretion of sIgA and the density of IgA^+^ plasma cells in the lamina propria were reduced by approximately 50% (73). This reduced secretion was unrelated to the Crohn’s Disease Activity Index (CDAI), suggesting that it may not be secondary to acute inflammatory damage but rather an intrinsic characteristic of CD patients. In contrast, sIgA secretion and IgA plasma cell density in this region were completely normal in UC patients. Furthermore, Preisker et al. found that IgA levels in intestinal tissue from CD patients in remission did not differ significantly from those of healthy controls, but fecal IgA levels were significantly reduced, accompanied by elevated local IgM levels in the ileum (74). The authors propose that this phenomenon results from primary IgA plasma cell induction or CSR defects in the intestines of CD patients in remission, leading to the local pathological retention of IgM^+^ B cells that have failed to complete the conversion. The reduction in protective sIgA in non-lesional areas weakens the intestine’s antigen-exclusion capacity, making luminal antigens more likely to penetrate the submucosal layer, which may create conditions for subsequent pathological class switching. In summary, conducting staging studies in conjunction with IBD disease activity is of great significance for elucidating abnormal changes in IgA CSR.

It is worth noting that in the healthy gut, a substantial proportion of IgA is not derived directly from IgM but instead requires a “sequential switching” process via an IgM→IgG1→IgA pathway (50). This intermediate stage is essential for the affinity maturation of antibodies. In IBD, this conversion pathway may become stalled at the IgG1 stage. This leads to the accumulation of IgG1 in the lamina propria, exacerbating local inflammation, while simultaneously reducing the production of downstream protective SIgA, thereby compromising mucosal defense. A recent *in vitro* experiment supports this hypothesis. The study demonstrated that even when removed from the *in vivo* inflammatory environment, converted memory B cells from CD patients still exhibit intrinsic differentiation defects when stimulated with IL-6 and APRIL *in vitro*. The upregulation of their maturation markers, CD138 and TACI, is impaired, leading to significantly reduced expression of J-chains and IGHA2 (encoding the IgA2 heavy chain) in differentiated plasma cells. This selectively weakens the secretory capacity of dimeric IgA, while IgG production remains largely unaffected (72). This suggests the presence of an intrinsic, program-specific barrier during the differentiation of memory B cells into mature plasma cells. Therefore, further elucidation of this developmental bottleneck may provide new insights into the abnormal patterns of B-cell isogenic conversion and recombination in IBD.

### 5.2 Abnormal intestinal IgG CSR in IBD

Upregulated secretion of IgM, IgA, and IgG is one of the characteristics of IBD. Scott MG et al. found that intestinal lamina propria mononuclear cells isolated from IBD patients spontaneously secreted higher levels of total IgG compared to controls (75). Subsequently, Rüthlein J et al. also observed that intestinal mononuclear cells from IBD patients produced higher levels of IgG, especially IgG1, than control subjects (76). Notably, IgG levels correlated with the degree of localized inflammation, even in minimally inflamed mucosa. Consistent with these findings, paired immunofluorescence staining of rectal mucosa showed a significantly higher proportion of IgG1^+^ cells in UC patients compared to controls, whereas the proportion of IgG2^+^ cells was markedly reduced (77). Recent studies using single-cell sequencing and flow cytometry have also confirmed this phenomenon. In the intestinal lamina propria of patients with active UC, both the number of IgG^+^ plasma cells and the IgG/IgA ratio are significantly elevated. Clonal profile analysis indicates that, among all IgG^+^ clones, the proportion of the pro-inflammatory IgG1 subtype is markedly increased, rising from 40% in healthy controls to 72.1% in UC patients (78). Despite low levels of IgG4 in humans, the number of IgG4^+^ plasma cells in the intestinal mucosa of UC patients showed positive correlation with endoscopic and pathological activity (79). Locally elevated IgG in the gut may contribute to intestinal inflammation and indirectly promote fibrosis. In UC, intestinal macrophages expressing Fcγ receptors are more readily activated by IgG, leading to increased IL-1β production that subsequently stimulates Th17 activation and neutrophil recruitment (80). Recently, it has been shown that IgG4^+^ plasma cells are associated with intestinal stenosis in patients with CD. These IgG4^+^ plasma cells may activate fibroblasts and myofibroblasts through platelet-derived growth factor secretion, leading to uncontrolled intestinal fibrosis. In conclusion, increased levels of IgG in the IBD intestine are associated with inflammation, and understanding the dysregulation of IgG CSR could facilitate IBD treatment.

Recently, a B-cell subset expressing T-bet and producing IgG has garnered significant attention due to its presence in several autoimmune diseases such as multiple sclerosis and systemic lupus erythematosus (SLE) (81, 82). In a mouse model of lupus, B-cell-specific T-bet deficiency results in reduced switching to IgG2a and, to a lesser extent, impaired switching to IgG2b and IgG3 (83). An increased frequency of T-bet^+^ B cells has been reported in the intestinal mucosa of CD patients, and their presence correlates with enhanced disease severity, suggesting a pro-inflammatory role in CD pathogenesis (84). These atypical T-bet^+^ B cells were predominantly IgG^+^ cells, indicating that cytokines driving T-bet upregulation may be therapeutic targets in IBD, including IFN-γ, IL-12, and IL-18 (85). Related transcriptomic studies have also found that both expanded naive B cells and IgG^+^ plasma cells in the intestines of UC patients exhibit significant expression of genes associated with the IFN response (78). This suggests that cytokines such as IFN-γ may promote IgG CSR in the intestines of IBD patients. Furthermore, compared to healthy controls, IBD patients has more significant BAFF expression in the intestinal mucosa (86). Given that BAFF can induce IgG1 class switching in murine splenic B cells (37), BAFF may contribute to the increased IgG CSR in IBD. In addition to cytokines, studies have identified a population of peripheral T helper cells (TPH cells) at the margins of diseased mucosa in UC patients that highly express CXCL13 but lack CXCR5. These cells possess the ability to recruit naive B cells and can synergize with type 1 immunity to promote local IgG production (78).

The increased IgG CSR observed in the intestines of IBD patients may also be associated with impaired regulatory mechanisms. The interaction between Tfh cells and Tfr cells plays a critical role in both the induction and regulation of antibody production. In peripheral circulation, Tfr cells from both healthy controls and UC patients can suppress Tfh-mediated IgM secretion and IgG class switching (69). However, the suppressive capacity of Tfr cells from UC patients is significantly diminished compared to those from healthy controls. Further study revealed that Tfr cells from UC patients had lower expression of CTLA-4 (69). CTLA-4 is essential for Tfr-mediated suppression, as CTLA-4 blockade virtually eliminated Tfr-mediated IgM inhibition and significantly reduced Tfr-mediated IgG inhibition (69). Although this study exclusively utilized peripheral blood samples, dysregulation of Tfh and Tfr cell function may represent one mechanism underlying enhanced intestinal IgG CSR in IBD.

Notably, this abnormally enhanced local intestinal IgG CSR process not only targets the gut microbiota but may also promote the production of various specific autoantibodies, thereby disrupting immune tolerance. In UC, the most common autoantibodies are atypical anti-neutrophil cytoplasmic antibodies (a-ANCAs) (87), which are detectable in approximately 30%–80% of UC patients and may contribute to tissue damage by activating neutrophils. Anti-goblet cell antibodies (GAB) and anti-integrin αvβ6 antibodies have also been observed in UC patients (78, 87). GAB primarily impairs intestinal mucus layer secretion by interfering with goblet cell function, whereas anti-integrin αvβ6 antibodies directly disrupt the integrity of the intestinal epithelial barrier by blocking its binding to fibronectin and inhibiting TGF-β. In CD, autoantibodies primarily target pancreatic exocrine cells, presenting as highly specific anti-pancreatic antibodies (PAB) (88). PAB mainly targets glycoprotein 2 (GP2). Studies show that the GP2 splice variant GP2#4 is specifically expressed on the surface of M cells in the terminal ileum and L cells in the colon (89). Under normal conditions, GP2#4 binds to the FimH protein on the bacterial surface, transporting bacteria downstream to initiate mucosal immune clearance. However, under CD pathological conditions, anti-GP2 autoantibodies (i.e., PAB) block this binding. Consequently, bacteria such as adherent-invasive *Escherichia coli* cannot be normally cleared. Instead, they adhere to and invade intestinal epithelial cells, exacerbating mucosal inflammation (89, 90). Additionally, neutralizing anti-IL-10 autoantibodies are present in some IBD patients (91). Most of these autoantibodies belong to the IgG isotype, and their production depends on the process of B cells CSR. In IBD, a shift in the class-switch direction toward IgG may promote the generation of pathogenic autoantibodies. The presence of autoantibodies can also form a vicious cycle with abnormal CSR: autoantibodies damage the intestinal mucosal barrier by activating complement or recruiting inflammatory cells, releasing more autoantigens, which further stimulate B-cell activation and maintain the abnormal class-switch direction.

In summary, pro-inflammatory IgG antibody (including IgG antibodies directed against pathogenic microorganisms and self-antigens) levels are elevated in the IBD gut, and the imbalance between promoting and inhibitory factors of IgG CSR plays a pivotal role in the development of intestinal inflammation. Reducing intestinal IgG CSR may be a potential therapeutic strategy for IBD management.

The “IgA-IgG CSR imbalance” in the gut is a key manifestation of humoral immune dysfunction in IBD, primarily involving three aspects. First, regarding cell fate selection, locally elevated pro-inflammatory factors (such as IL-4, IL-21, and IFN-γ) suppress physiological IgA class-switch signals, leading to increased IgG CSR. Second, regarding the class-switch pathway, the normal sequential IgM→IgG1→IgA class-switch mechanism is stalled by the inflammatory environment, causing IgG1^+^ B cells, which serve as transitional intermediates, to accumulate in the lamina propria and secrete pro-inflammatory antibodies. Finally, regarding subclass effects, there is an abnormal increase in the strongly pro-inflammatory IgG1 subclass, while the switch to the protective IgA2 subclass is impaired. Disruptions in CSR in IBD not only exacerbate mucosal damage, but may also induce genomic instability in epithelial cells, providing a potential basis for the development of inflammation-associated colorectal cancer.

### 5.3 Bowel inflammation-associated colorectal cancer with AID

Patients with IBD are at increased risk of colorectal cancer, especially those with UC (92). Under physiological conditions, AID contributes to antibody gene diversification in activated B cells through somatic hypermutation and CSR of immunoglobulin genes. However, during intestinal inflammation, abnormally elevated AID-induced gene mutations may contribute to the increased risk of colorectal cancer in patients with IBD. A study found that endogenous AID expression was significantly higher in IBD model mice (IL-10^−/−^) compared to wild-type mice after 20 weeks (93). Genetic ablation of AID reduces both the somatic mutation frequency of the tumor suppressor gene Trp53 in colonic mucosa and the development of colitis-associated colorectal cancer (93). Laura P. Hale et al. constructed a novel colitis model - “TIA” mice (94). Mice deficient in both Tnf and IL-10 (termed T/I mice) spontaneously develop moderate-to-severe colitis at 4–6 weeks, exhibiting clinical and histopathological patterns resembling human UC. TIA mice (Tnf^-/-^, IL-10^-/-^, Aicda^-/-^) with additional AID deficiency also develop spontaneous UC-like colitis shortly after birth, with inflammatory severity similar to T/I mice (95). However, TIA mice show significantly fewer colorectal tumor lesions than T/I mice, demonstrating that Aicda deletion attenuates inflammation-associated colorectal tumorigenesis without preventing inflammatory tumor development. Importantly, the TIA mouse model constructed by Laura P Hale et al. will be of great help in our future studies of the role of CSR in IBD. All of these studies support the critical role of AID pathways in the development of inflammation-associated cancers, which may lead to a new strategy: inhibiting the development of colorectal cancer in IBD patients by targeting AID.

### 5.4 IBD and vaccination

Considering the dysregulated class switching in B cells, the efficacy of vaccinations in IBD patients may be compromised, particularly for vaccines that activate B cells, such as the 23-valent pneumococcal polysaccharide vaccine. Composed of polysaccharides, this vaccine contains T cell-independent antigens that can directly target B cells, which subsequently produce protective antibodies (IgG and IgM) against *Streptococcus pneumoniae* (96). The increased IgG CSR observed in the intestinal mucosa of IBD patients may disrupt the immune pathways activated by vaccination, potentially exerting negative effects on IBD progression. Currently, available data regarding the effectiveness of various vaccines in IBD patients remain insufficient, warranting heightened caution when administering vaccinations to IBD patients.

## 6 CSR as a potential therapeutic target for IBD

As mentioned above, IgA plays a crucial role in defending against pathogens and maintaining intestinal homeostasis. In contrast, pro-inflammatory IgG levels are elevated in the intestines of IBD patients. Therefore, targeting CSR is a promising therapeutic strategy for IBD. Enhancing intestinal IgA CSR or suppressing IgG CSR may ameliorate intestinal inflammation in IBD.

### 6.1 Enhance intestinal IgA CSR

Multiple studies have found that oral administration of the monoclonal IgA antibody W27 can modulate the composition of the intestinal microbiota in mice to achieve symbiotic balance, which exhibits therapeutic effects against DSS-induced colitis (27, 97). In experimental mouse models of colitis, exogenous IgA supplementation has been shown to ameliorate colonic inflammation, suggesting that increasing intestinal IgA levels may represent a viable therapeutic strategy for IBD.

As previously discussed, many cytokines participate in the regulation of CSR. Increasing the levels of cytokines in the gut that promote the IgA CSR may be a potential therapeutic strategy for IBD. Retinoic acid, a metabolite of vitamin A, enhances IgA production by stimulating DCs to release active TGF-β, suggesting that dietary vitamin A supplementation could potentially increase intestinal IgA levels and reduce gut inflammation. Regulatory B cells (Bregs), a specialized B-cell subset with immunosuppressive functions, mediate immune suppression through the secretion of IL-10 and TGF-β (98). Given that both TGF-β and IL-10 have been shown to support IgA CSR (66), intestinal Bregs may facilitate IgA CSR, although this specific relationship has not yet been reported in the literature. The interaction between intestinal Bregs and CSR deserves further investigation. Additionally, HIF-1α promotes IgA CSR by increasing histone acetylation in the Sα region through acetyl-CoA. Increasing HIF-1α levels or supplementation with acetyl-CoA may enhance intestinal IgA secretion and has potential for alleviating intestinal inflammation (62).

Bacteroides species have been demonstrated to enhance IgA class switching and production in murine intestinal B cells, while soluble dietary fibers increase the abundance of Bacteroides (including B. acidifaciens) and promote IgA production (60). Consequently, soluble dietary fibers may improve intestinal immune function, thereby protecting against enteric pathogens and reducing the incidence of IBD.

Furthermore, high IgA coating selectively identifies pro-inflammatory microbiota in both murine and human intestines (29). The host’s IgA response to gut microbiota may serve as a biomarker to identify microbial taxa that preferentially influence disease susceptibility or severity. Targeted elimination or replacement of these disease-driving microbiota could potentially attenuate, reverse, or even prevent disease progression (29).

### 6.2 Suppress intestinal IgG CSR

Compared to healthy controls, IBD patients had significantly higher BAFF expression in the intestinal mucosa (86). Considering that BAFF can induce IgG1 CSR in murine splenic B cells (58), BAFF may contribute to the elevated IgG CSR in IBD. Targeting BAFF signaling may therefore reduce intestinal IgG CSR. Notably, given BAFF’s established role in systemic autoimmune diseases such as SLE and rheumatoid arthritis, several BAFF-targeted therapies have already been commercialized. Current BAFF-targeting agents under clinical investigation include belimumab (99), atacicept (100), tabalumab (101), and blisibimod (102). Inhibiting abnormal humoral immunity in the gut by blocking BAFF may represent a promising therapeutic strategy for IBD. Whether modulating pro-inflammatory factors can indirectly influence specific autoantibody levels remains a subject of debate in clinical research. Derer et al. found that patients with ileocecal Crohn’s disease receiving anti-TNF-α therapy exhibited a significant decrease in anti-GP2 autoantibody levels in peripheral blood (89). This suggests that conventional anti-TNF therapy may, to some extent, attenuate the abnormal humoral immune response directed against intestinal receptors by reducing the titers of specific antibodies. However, this regulatory effect exhibits significant heterogeneity across different study cohorts. Michaels et al. found that anti-GP2 autoantibody titers in CD patients remained highly stable during long-term follow-up with a median duration of 426 days and did not rapidly decline during anti-inflammatory treatment with anti-TNF-α agents (90). These conflicting results may be related to the location of lesions and the stage of disease progression in patients, or they may be influenced by the sensitivity of different detection methods. Future multicenter prospective cohort studies using a standardized detection platform are necessary to determine whether blocking cytokines can reduce autoantibody levels in IBD patients. A deeper understanding of this regulatory mechanism holds promise for developing new therapeutic strategies for IBD.

### 6.3 Limitations and future perspectives

Although modulating the CSR pathway to correct intestinal “IgA-IgG imbalance” holds potential therapeutic value, translating this into a clinical strategy currently faces numerous limitations. On the one hand, most of the current molecular evidence regarding the CSR pathway and its microenvironment-induced signaling is based on mouse models, and it remains to be verified whether these findings can be fully extrapolated to the complex intestinal mucosal environment of human IBD patients. On the other hand, direct intervention in cytokines is highly complex and may lead to unpredictable and potentially harmful consequences. IL-21 can promote IgG1/IgG2b CSR (65). Recently, interference with IL-21 signaling has been proposed as a therapeutic approach for various autoimmune diseases, including SLE (103) and rheumatoid disorders (104). However, Elisabeth Salzer et al. identified an IL-21 gene mutation causing IL-21 deficiency in a patient with early-onset IBD. While this deficiency led to reduced circulating CD19^+^ B cell numbers, it did not impair B cell activation or CSR capability (105). This finding establishes IL-21 deficiency as a novel cause of primary immunodeficiency and early-onset IBD, highlighting the need for caution when employing cytokine-targeted therapies for autoimmune diseases like IBD. Targeting cytokines to modulate CSR may inadvertently disrupt other immune processes. Furthermore, certain cytokines exhibit pleiotropic effects on B cell isotype switching - for instance, both BAFF and APRIL appear to simultaneously promote IgG and IgA CSR. Consequently, caution is warranted when using cytokine-targeted therapies for IBD.

In addition to direct therapeutic interventions, utilizing B-cell lineage characteristics for precise diagnosis and disease stratification is also an important area for translational research. For example, Shen et al. achieved a high accuracy of AUC = 0.963 in CD diagnosis using a convolutional neural network (CNN) classifier constructed from 13 key B-cell genes and an immune infiltration matrix (9). This suggests that in the future, it may be possible to combine the antibody profiles of intestinal mucosal B cells or the expression profiles of CSR-related genes with deep learning models, which could help guide clinical decision-making.

## 7 Conclusion

The intestine represents one of the largest mucosal surfaces in the human body. Intestinal mucosal immunity must simultaneously generate protective immunity against pathogens while maintaining tolerance to harmless food antigens and commensal microorganisms, posing a significant challenge to the gut immune system. In healthy intestines, the predominant antibody IgA plays a dual role: it provides protection by binding and “coating” pathogenic organisms to exclude them from the intestinal surface or inhibit their growth, while also binding beneficial microbes to anchor them within the mucus layer, thereby maintaining intestinal homeostasis. IgG in healthy intestines also contributes to protection by reducing pathogenic colonization and preventing allergic intolerance. However, increased IgG levels in IBD may excessively activate FcγRs or stimulate Th responses, leading to intestinal inflammation. IBD, a chronic relapsing inflammatory bowel disorder, significantly impacts patients’ quality of life. Given that most patients show intolerance to conventional therapies, enhancing intestinal IgA CSR or reducing IgG CSR may offer promising therapeutic alternatives. Nevertheless, targeting CSR to ameliorate intestinal inflammation in IBD faces several challenges. First, most current CSR studies are conducted *in vitro*, while *in vivo* conditions are considerably more complex. Whether specific signals (including cytokines and microbial factors) can induce CSR toward particular antibody isotypes in the presence of other immune cells, as observed *in vitro*, requires further investigation. Additionally, many signaling pathways exhibit pleiotropic effects, and the potential consequences of blocking these signals on other immune functions remain unclear. These critical questions warrant further exploration.
